# Education to improve timeliness of shingles diagnosis in primary care: a cluster randomised study within a trial with nested qualitative study

**DOI:** 10.3399/BJGP.2023.0477

**Published:** 2024-08-20

**Authors:** Elizabeth Lovegrove, Stephanie J MacNeill, Yumeng Liu, Sophie Rees, Jonathan Banks, Robert Johnson, Matthew J Ridd

**Affiliations:** Primary Care Research Centre, University of Southampton, Southampton.; Bristol Trials Centre, University of Bristol, Bristol.; Bristol Trials Centre, University of Bristol, Bristol.; Bristol Trials Centre, University of Bristol, Bristol.; National Institute for Health and Care Research Applied Research Collaboration West, Bristol; Bristol Medical School, University of Bristol, Bristol.; Bristol Medical School, University of Bristol, Bristol.; Bristol Medical School, University of Bristol, Bristol.

**Keywords:** diagnosis, general practice, herpes zoster, interprofessional education

## Abstract

**Background:**

Herpes zoster (shingles) is normally diagnosed clinically. Timely diagnosis is important so that antiviral treatment can be started soon after rash onset.

**Aim:**

To assess whether a practice-level educational intervention, aimed at non-clinical patient-facing staff, improves the timely assessment of patients with shingles.

**Design and setting:**

This was a cluster randomised study within a trial (SWAT) with nested qualitative study in general practices in England.

**Method:**

Practices were cluster randomised 1:1, stratified by centre and minimised by practice list size and Index of Multiple Deprivation score. Intervention practices were sent educational materials, highlighting the common presenting features of shingles and what action to take if suspected. The primary and secondary outcomes were the mean proportion of patients per practice seen within 72 and 144 h of rash onset, respectively. Comparison between groups was conducted using linear regression, adjusting for randomisation variables. Semi-structured interviews with practice staff in intervention practices explored views and opinions about the intervention.

**Results:**

In total, 67 practices were enrolled; 34 randomised to the intervention and 33 to the control. The mean difference in proportion of patients seen within 72 and 144 h was −0.132 (95% confidence interval [CI] = −0.308 to 0.043) and −0.039 (95% CI = −0.158 to 0.080), respectively. In intervention practices, 90.9% reported distributing the educational materials; however, engagement with these was suboptimal. Twelve participants were interviewed, and the poster component of the intervention was said to be easiest to implement.

**Conclusion:**

Our educational intervention did not improve the timely assessment of patients with shingles. This may be the result of poor intervention engagement.

## Introduction

Approximately one in four people will develop herpes zoster (‘shingles’) in their lifetime, and prevalence and severity increases with age.^1^ It occurs because of reactivation of the varicella zoster virus in the dorsal root, cranial, or sensory ganglion, often decades after the initial infection.^2^ Shingles is usually diagnosed in primary care based on the characteristic painful, blistering dermatomal rash that occurs on one side of the body. This is often preceded by a prodrome of malaise, fever, pain in the dermatome where the rash subsequently appears, and paraesthesia or dysaesthesia.^2^ Persistent, severe pain can follow (postherpetic neuralgia [PHN]), which significantly affects quality of life.

Clinical guidelines recommend that oral antiviral treatments are started within 72 h of rash onset for people who are immunocompromised, whose shingles affects their head, neck, or limbs, and those who have a moderate-to-severe rash or moderate-to-severe pain.^2^^–^4 Furthermore, the National Institute for Health and Care Excellence (NICE) advise that antiviral treatment should be considered for patients presenting within 72 h and who are aged >50 years, as this age group is at increased risk of PHN.^2^ In routine clinical practice, antivirals are often started up to 1 week after rash onset, partly because patients are often not being diagnosed within the 72 h window.

In UK general practices, non-clinical staff often act as gatekeepers to clinician appointments. Receptionists with little or no clinical training routinely seek the reason for the appointment as a form of triage.^5^ We sought to determine if a practice-based educational intervention, aimed at improving the knowledge of non-clinical staff, improved the timely diagnosis of new-onset shingles.

## Method

### Trial design

This cluster randomised study within a trial (SWAT) was hosted by the AmiTriptyline for the prevention of post HErpetic NeuralgiA (ATHENA) trial. ATHENA is a multicentre, individually randomised, placebo-controlled superiority trial (ISRCTN: 14490832) that aims to find out if prophylactic low-dose amitriptyline can prevent PHN in patients aged >50 years diagnosed with shingles.

**Table:** How this fits in

The timely assessment of shingles as soon as possible after rash onset is important for the effectiveness of antiviral medication. Reception staff are gatekeepers to appointments in primary care. A practice-level educational intervention, aimed at non-clinical staff, was not found to improve the proportion of patients diagnosed within 72 or 144 h of shingles rash onset, possibly as a result of limited engagement with educational materials.

All practices enrolled in the host ATHENA trial during the first 6 months of participant recruitment, with the exception of one, participated in the SWAT. GP practices were recruited from West of England, Wessex, Thames Valley and South Midlands, and South West Peninsula National Institute for Health and Care Research (NIHR) Clinical Research Networks (CRNs). Results for GP practices from the South West Peninsula CRN area are reported under the West of England CRN. The SWAT ran between 11 April and 10 October 2022, with the final practice randomised on 3 August 2022. Depending on the date of enrolment, practices received intervention or control materials for varying periods of time (see Supplementary Figure S1, and Supplementary Tables S1 and S2).

If practices operated across multiple sites and shared reception staff, they were randomised as a single unit to avoid any contamination. Participants in intervention practices included all patient-facing staff (receptionist or administrative staff, and clinical staff).

### Intervention

In addition to standard patient-facing ATHENA posters, intervention practices were sent:

- five posters per practice site to display in staff areas (see Supplementary Figure S2a);
- desktop backgrounds to be uploaded onto all computers of patient-facing staff (see Supplementary Figure S2b); and
- a link to a 1-min video hosted on YouTube (https://www.youtube.com/watch?v=Kx7TrYfusik).

**Figure 1.**
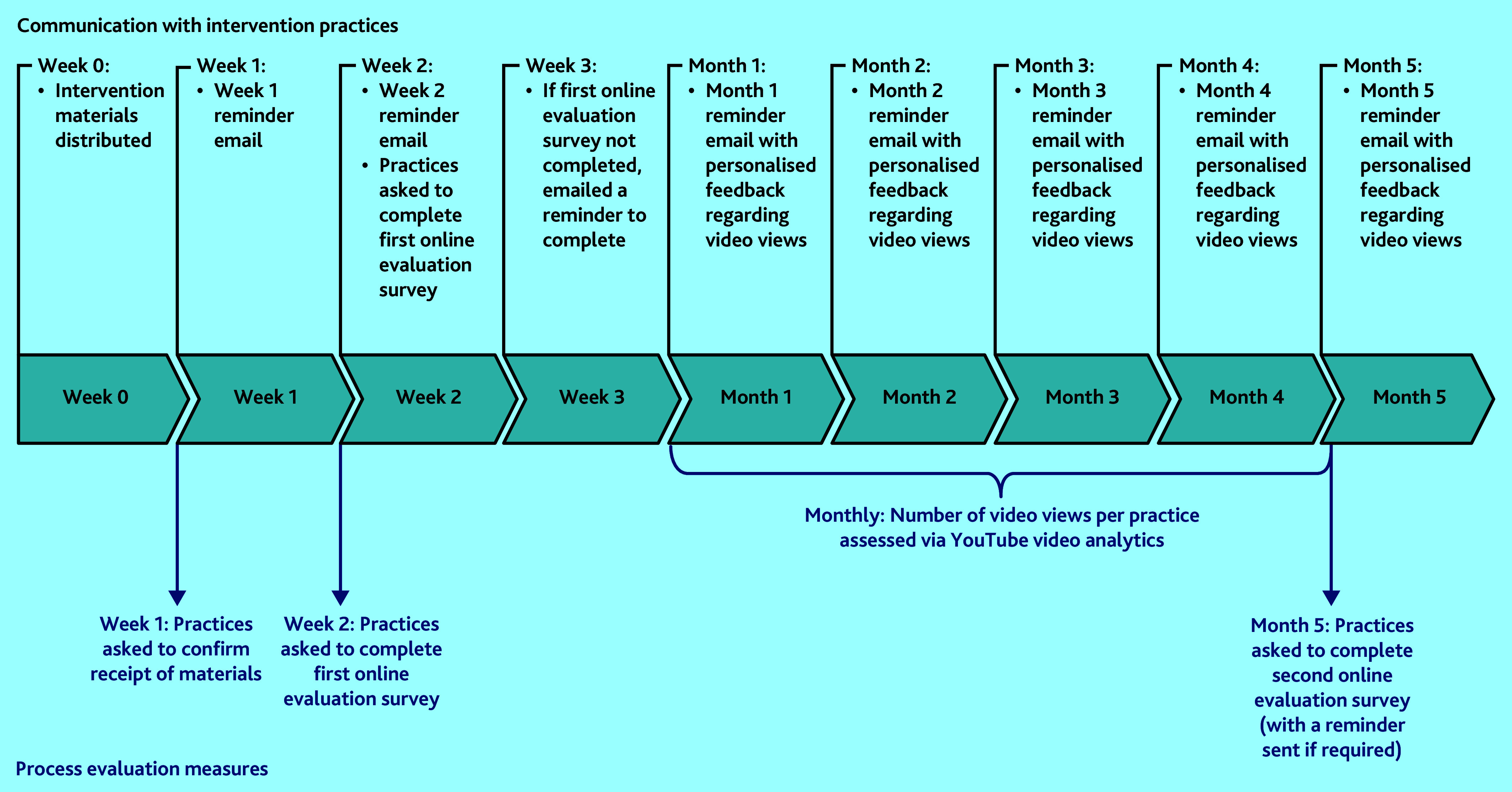
Timeline of all contacts (all via email) with intervention practices (black text) and methods used to evaluate use of the intervention materials, that is, process evaluation measures (blue text). Both evaluation surveys asked practices to confirm the number of posters and desktop backgrounds on display. Video usage data were obtained using YouTube analytics software.

Research leads were subsequently emailed at week 1, 2, and then on a monthly basis to remind them about the study until the end of the SWAT, and personalised feedback on video engagement was also provided monthly (see Figure 1 and Supplementary Information S1).

All three educational components included information about shingles symptoms and signs, the importance of early recognition, what action to take if suspected, and how quickly to take this action in the form of a three-phrase instruction: ‘Think shingles, get shingles seen, treat shingles soon’. This phrase was based on the ‘COM-B’ model and principles recommended by the British Psychological Society: people’s capabilities (C), opportunities (O), and motivations (M) need to be optimised, to result in a behaviour (B) change.^6^^–^8

### Comparator

All practices were asked to display the ATHENA trial patient-facing waiting room poster (see Supplementary Figure S3).^9^

### Outcomes

The primary outcome was the proportion of patients seen within 72 h since their rash onset. The secondary outcome was the proportion of patients seen within 144 h since their rash onset.

Time since shingles rash onset was collected on the host ATHENA trial clinician screening and referral form. Clinicians were encouraged to complete this form, which included a question on the number of days since rash onset, for all patients aged ≥50 years diagnosed with shingles, even if not eligible for the trial.

### Process measures

The design of our implementation evaluation was based on two recent systematic reviews that recommended electronic and personalised reminder emails as the most effective strategies to also improve engagement.^10^^,^^11^ Therefore, implementation of the intervention materials was assessed by (Figure 1):

- requesting practices provide email confirmation that they had received the materials;
- asking practices to complete an online survey regarding the number of posters and desktop backgrounds displayed at week 2 and in the final month of the SWAT; and
- YouTube analytics software was used to evaluate practice engagement with the animation video monthly.

A simple implementation score (zero to three) was calculated for each practice, with one point for engagement with each of the intervention material modalities (poster, desktop background, and animation video).

### Sample size

Study sample size was dependent on the host ATHENA trial, therefore in keeping with agreed SWAT methodology, no formal sample size calculation was performed.^12^^,^^13^ However, assuming 60% of patients at a GP practice are seen within 72 h of rash onset in usual care and a standard deviation (SD) of 25, recruiting 60 practices in total would allow the study to detect an absolute increase of 20% (thus, 80% of patients at intervention practices being seen within 72 h of rash onset) with 86% power.

### Randomisation, allocation, and masking

The first author enrolled practices, the third author independently randomly assigned (1:1, stratified by CRN and minimised on practice list size and Index of Multiple Deprivation [IMD] for middle layer super output areas [MSOAs] in England) the practice to intervention or control, and the first author communicated with each practice their SWAT allocation. The IMD score was generated using the population-weighted average score of IMD 2019 scores for all the lower layer super output areas within the MSOA that each practice is located in.^14^ Allocation concealment for the clusters was achieved by a central randomisation program, where the allocation of each cluster was automatically assigned by running a pre-validated Stata program with a seed being pre-specified, based on all the randomisation variables. The second, sixth, and senior author were masked, and the first, third, fourth, and fifth authors and participating practices were not masked.

### Analysis

Analyses for this SWAT were on a modified intention-to-treat basis, using Stata SE (version 17). The primary and secondary outcomes were analysed using a linear regression model with treatment arm and all variables used in the randomisation as covariates. The coefficient effect comparing the intervention and control group with corresponding 95% confidence intervals (CIs) and *P*-values are reported. A sensitivity analysis to adjust for the duration of follow-up (the number of days between communication of practice allocation and 10 October 2022) at the individual practice was not required as the length of follow-up was well balanced by arm (Table 1).

**Table 1.** Baseline characteristics of participating practices

**Characteristic**	**Intervention**	**Control**	**All**
**Total participating practices, *n***	34	33	67

**Practices with list size >10 000, *n* (%)**	24 (70.6)	23 (69.7)	47 (70.1)

**List size, mean (SD)**	18 795.0 (16 738.9)	13 542.9 (7182.1)	16 208.1 (13 118.9)

**Practices within each CRN area, *n* (%)**			
West of England	23 (67.6)	23 (69.7)	46 (68.7)
Wessex	5 (14.7)	5 (15.2)	10 (14.9)
Thames Valley and South Midlands	6 (17.6)	5 (15.2)	11 (16.4)

**IMD score (at practice level), median (IQR)**	12.68 (9.19–18.85)	12.31 (8.87–17.30)	12.49 (8.9–18.9)

**Days enrolled in SWAT, mean (SD)**	129.0 (44.7)	132.4 (43.2)	130.7 (43.7)

**Patients screened, mean (SD)^a^**	6.7 (6.5)	6.5 (5.4)	6.6 (6.0)

**Practices that screened ≥1 patient, *n* (%)**	30 (88.2)	26 (78.8)	56 (83.6)

**Age of patients screened, years, mean (SD)^a^**	67.0 (5.5)	69.2 (6.8)	68.0 (6.2)

**Female patients screened, %, mean (SD)^a^**	73.9 (26.8)	57.2 (24.2)	66.2 (26.8)

a *Mean of those practices screening ≥1 patient. CRN = Clinical Research Network. IMD = Index of Multiple Deprivation. IQR = interquartile range. SD = standard deviation. SWAT = study within a trial.*

Descriptive statistics were performed regarding practice engagement, with each intervention component and findings presented as counts, proportions, and means unless otherwise stated.

### Nested qualitative study

Semi-structured interviews were conducted with practice staff (GPs, non-GP health professionals, and administrative/managerial staff) to explore views regarding the main trial and the SWAT intervention. We sought to interview 10–12 participants from intervention practices. Staff who expressed an interest were sent an information sheet and gave verbal consent to taking part. Interviews took place online via Microsoft Teams and were audio-recorded and transcribed verbatim. Transcripts were checked for accuracy and pseudonymised.

We used the framework method to analyse the data, which was conducted rapidly, concurrent with data collection. We created a framework to map data from the interviews onto a predefined framework based on the questions:^15^^,^^16^

- what challenges and facilitators did practice staff encounter when implementing the intervention? And
- what are the views of staff with regards to the impact of the intervention in their practice?

Although the framework was pre-defined, we allowed space to capture data that did not fit in these questions.

### Patient and public involvement

The views of a patient advisory group were sought in the design of the host ATHENA trial and continued during trial delivery. SWAT design, intervention materials, and results were discussed with this group.

### Ethics

All GP surgeries enrolled in the main ATHENA trial also consented to being enrolled in the SWAT. All control practices were offered the intervention animation after the 6-month evaluation period.

### Study registration

The study protocol was registered with The Northern Ireland Hub for Trials Methodology Research and is listed in their SWAT repository (SWAT ID: 173).^17^ The host trial protocol is available at https://www.bristol.ac.uk/athena-study.^9^

## Results

### GP practice recruitment

**Figure 2.**
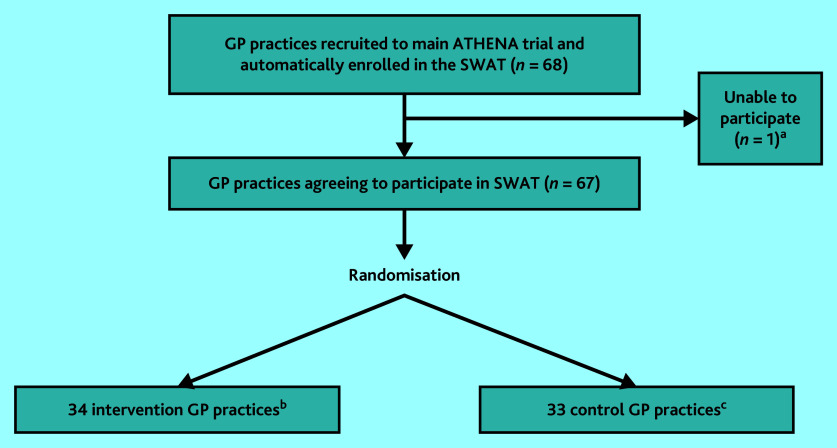
CONSORT flow diagram for GP practices enrolled in the SWAT. ^a^One GP practice was unable to participate in the SWAT because of absence of the GP Principal Investigator at the time of randomisation. ^b^ Thirty-four intervention practices were made up of 20 individual practice sites, six groups of practices that were randomised as a whole group, and eight practice sites that were part of a larger practice group but randomised individually for the purposes of the SWAT. ^c^The 33 control practices were made up of 20 individual practice sites, four groups of practices that were randomised as a whole group, and nine individual practice sites that were part of a larger practice group but randomised individually for the purposes of the SWAT. SWAT = study within a trial.

As seen in Figure 2, 68 practices were eligible to take part in the SWAT but one was unable to take part; 34 practices were randomised to the intervention and 33 practices to the control. Supplementary Figure S1 shows recruitment of GP practices during the SWAT.

The baseline characteristics of participating SWAT practices and patients who were screened for enrolment in the host ATHENA trial are shown in Table 1; 56 (83.6%) practices screened ≥1 patient. The two arms were balanced with respect to CRN area, practice list size, IMD scores (Table 1), and duration of follow-up (see Supplementary Tables S1 and S2). For additional details on participating practices by allocation, see Supplementary Table S2.

### Primary and secondary outcomes

Table 2 shows the number of days since rash onset by practice allocation for all practices. For the primary outcome, there was weak evidence of any difference in the mean proportion of patients seen within 72 h of rash onset between intervention (43.6%) and control group (57.2%) practices (−13.2%, 95% CI = −30.8 to 4.3, *P* = 0.135) (Table 3). Similarly, for the secondary outcome, there was no evidence of any difference (82.4% versus 86.7%, −3.9%, 95% CI = −15.8 to 8.0, *P* = 0.514).

**Table 2.** Days since rash onset by practice allocation of 366 screened patients with complete data

**Days since rash onset**	**Patients, *n* (%)**		
	**Intervention**	**Control**	**All**
0	3 (1.5)	9 (5.4)	12 (3.3)
1	27 (13.6)	29 (17.3)	56 (15.3)
2	42 (21.2)	36 (21.4)	78 (21.3)
3	30 (15.2)	24 (14.3)	54 (14.8)
4	28 (14.1)	16 (9.5)	44 (12.0)
5	24 (12.1)	13 (7.7)	37 (10.1)
6	8 (4.0)	12 (7.1)	20 (5.5)
>6	36 (18.2)	29 (17.3)	65 (17.8)
Total	198 (100.0)	168 (100.0)	366 (100.0)

**Table 3.** Primary and secondary outcomes

**Outcome**	**Intervention**	**Control**	**Difference (95% CI)**	***P*-value**
Practices referring ≥1 patient, *n*	30	26	—	—
Proportion of patients seen within 72 h of rash onset, mean (SD)	0.436 (0.319)	0.572 (0.309)	−0.132 (−0.308 to 0.043)	0.135
Proportion of patients seen within 144 h of rash onset, mean (SD)	0.824 (0.244)	0.867 (0.181)	−0.039 (−0.158 to 0.080)	0.514

*SD = standard deviation.*

No harms or unintended effects were reported in the intervention or control groups.

### Process evaluation

Of the 22 (64.7%) intervention practices that completed the 2-week online survey, 20 (90.9%) had distributed the intervention materials to staff. The mean percentage of posters displayed per practice was 76.3% and the mean percentage of receptionists’ computers displaying the desktop background was 31.0%. Of the 19 (55.9%) practices that completed the online survey in the final month of the SWAT, the mean percentage of posters displayed per practice was 81.1% and 31.4% of receptionists’ computers were displaying the desktop background (see Supplementary Tables S3 and S4).

The video was watched 296 times: 26 (76.5%) intervention practices watched the video at least once; of these, the mean number of video views per practice was 11 and the average percentage of the video duration viewed was 74.4%. (see Supplementary Table S5).

In total, 24 intervention practices completed the 2-week and/or final online surveys regarding poster and desktop background use, and therefore a composite implementation score could be calculated (see Supplementary Table S6). Most (*n* = 17, 70.8%) did not engage with the entire package of education materials (posters, desktop backgrounds, and videos). Of those practices that had screened ≥1 patient, there was a weak positive correlation between the implementation score and the primary outcome (correlation coefficient 0.31) and a weak negative correlation between composite score and the secondary outcome (correlation coefficient −0.18) (see Supplementary Figure S4).

### Qualitative findings

Twelve practice staff (five GPs; three research nurses [RNs]; two practice managers; one paramedic; and one pharmacist) from 10 intervention practices were interviewed; we attempted to recruit reception staff but were unsuccessful. Interviews lasted on average 28 min (range 22–39 min). The SWAT formed a part of the interview that was about the ATHENA trial in general.

We developed two main themes:

- challenges of implementing the intervention; and
- views on the impact of the intervention.

#### Challenges of implementing the intervention

Although most participants stated that the intervention materials had been implemented, there was uncertainty regarding engagement with them:

*‘I meant to go round and have a look. I’ve circulated it all but nobody has come back to me with any comments which may or may not be good* [laughs].*’*

(AQ022, GP)

In some practices, the IT system would not allow individuals to change their desktop background and there was a sense that the desktop backgrounds were not useful owing to them being hidden by windows when people are working. Furthermore, there was resistance from staff to the content being on permanent display, with pictures of dermatological conditions seen as unwelcome additions to workstations:

*‘The way that it’s set up with* [IT system]*, if you change a background, every time you log out it just defaults it back to the blue screen. And it’s to stop staff putting their own backgrounds on their desktops.* [Name] *who was our IT manager at the time, she took it to the CCG* [clinical commissioning group] *and they said basically you can’t do it.’*

(AQ027, RN)

*‘I think they wanted us to have a screen saver, desktop background, yeah, that’s not happened ’cos the receptionists think it’s disgusting first of all which puts them off their coffee, plus when you’re working you don’t really have your desktop … it’s not there, it’s not up so it’s not really a reminder because they’ve got the screens up, they’re working, they’re not really going to look at it and they all thought it was horrible* [laughs]*.’*

(AQ025, GP)

Overall, the posters were seen as the most useful, and these had been placed in various locations including patient waiting rooms, reception areas, staff toilets, and consulting rooms. Participants were unsure of the uptake of the animated video and sometimes of the desktop backgrounds.

#### Views on the impact of the intervention

Most participants felt that the SWAT intervention would not have an impact on the speed at which patients with potential shingles were seen:

*‘Our receptionists have a relatively low threshold for putting anything and everything on the duty list, and that’s the default … They’ve no triage role really.’*

(Q039, GP)

*‘I think we’ve got really good access for our patients so I think they* [patients] *would also go through the duty doctor or the clinician within a couple of days anyway.’*

(AQ022, GP)

There was also an acknowledgement that reception staff are already extremely busy, and there was a reluctance to ask any more of them:

*‘Our reception staff are all phenomenal, all of them, but we are 80 hours short of receptionists … the pressure they’re under is literally just constantly, constantly the phones they just don’t stop. When I first started there I used to quite often go and hang out in the phones room to chat, you just can’t do that anymore. So I think it’s just again another pressure that, you know, public facing members of primary care face.’*

(AQ027, RN)

*‘They are just overloaded at the moment … we’re very short staffed in reception and GPs.’*

(AQ026, RN)

A few participants felt that the intervention might make a difference in terms of potential participants for the main trial being flagged to the relevant staff:

*‘All the receptionists know about it, so basically anybody who phones in query shingles they’ll highlight it for me to have a look at and then I’ll just slot it into my surgery or if the clinician is seeing the patient I’ll see them afterwards, that’s the plan.’*

(AQ025, GP)

However, this was focused on making sure the research-active staff were aware of potential participants for the host ATHENA trial, rather than making sure they were booked in quicker for shingles diagnosis.

Practices adopted different recruitment strategies for the host ATHENA trial. In those that relied on periodic searches (rather than the electronic ATHENA trial ‘pop up’ that was triggered during consultations regarding shingles) to identify and follow-up recent shingles consultations, the SWAT intervention was not seen as relevant. This was because reception staff were not involved in identifying ATHENA participants, even though the intervention was intended to speed up all shingles diagnoses, and therefore increase the pool of potential participants for the ATHENA trial:

*‘It’s all good content, but it didn’t fit with our recruitment model.’*

(AQ036, research manager)

*‘It’s not gonna make the receptionist do anything different that would mean that I would suddenly find a patient.’*

(AQ033, RN)

This suggests that the purpose of the SWAT intervention was sometimes forgotten or misunderstood by practice staff.

In practices where the approach to ATHENA participant recruitment was to rely on GPs in the practice to screen and refer during consultations, the GPs’ capacity and high workload was seen as the limiting factor to recruitment:

*‘It’s the GPs. I don’t want to criticise because I just find this all the time, I just think they’re just so busy, it’s just that the workload is just astronomical and it’s just one more thing I think to think about, you know, and having to do.’*

(AQ027, RN)

The qualitative findings provide context and insight into the quantitative findings. On the whole, participants did not see the SWAT as an effective intervention. The desktop background was seen as ineffective, as it was hidden behind windows while people worked, and some care service IT systems did not support its implementation. The intervention was not seen as worthwhile by some practices because it was perceived to not fit with their participant identification strategies for the ATHENA trial. The SWAT intervention assumed that patients with shingles were not booked in immediately, but this did not appear to be the case. It also assumed that reception staff would triage based on the appearance of a rash, but we did not find evidence of this.

## Discussion

### Summary

Our educational intervention did not improve the proportion of patients with shingles seen within 72 or 144 h. This may be because the intervention was truly not effective, it was not implemented sufficiently, or we have incomplete/insufficient data. Our results show that of those practices that submitted data on engagement with the intervention, 70.8% did not engage with all materials that were distributed. The qualitative findings suggest practice staff did not perceive the intervention as effective, and with staff already facing high workload it was sometimes not considered worthwhile. The SWAT intervention did not fit with some of the chosen models of recruitment seen in some practices.

### Strengths and limitations

To our knowledge, this is the first SWAT in the UK to evaluate if an educational intervention, aimed at non-clinical reception staff, can reduce the time taken to diagnose shingles and also improve clinical trial recruitment.^17^ Our study also demonstrates the challenges in changing the behaviour of non-clinical reception staff who are often gatekeepers to clinician appointments; an under-researched area. We successfully undertook this SWAT in the context of a large, multicentre randomised clinical trial of an investigational medicinal product. We demonstrated that intervention implementation, monitoring, and outcome data collection can be undertaken remotely, albeit with variable levels of reported engagement and the following limitations.

The primary outcome was based on data collected when clinicians completed the screening and referral form for the main trial, even if the patient with shingles was ineligible or not interested in taking part. These data will be incomplete, and it is possible that differential completion/non-completion of this form between intervention and control practices may have affected our outcome.

We were reliant on practices being honest about their implementation of the posters and desktops backgrounds. YouTube analytics provided some independent, objective evidence of practice-specific engagement but it was not possible to differentiate between different staff members engaging with the animation video, using the same internet protocol address but at different times. This is likely considering most computers are shared among practice staff. Therefore, number of video views may not reflect the number of different individuals who watched the video and may be an overestimate of practice engagement with this resource.

The impact of our intervention may have been limited owing to suboptimal engagement with the intervention materials. Our qualitative findings provide some insight into possible reasons for this; difficulties with installation and visualisation of the desktop background, and the intervention materials were not felt to be relevant to non-clinical reception staff in some surgeries depending on their appointment booking system, thus reducing engagement.

As this study was undertaken as a SWAT, the number of clusters and sample size was determined by the number of sites and patients screened during the first 6 months of participant recruitment for the host trial, rather than a pre-specified target. We were not powered to detect a difference of <20%. Furthermore, the needs of the host ATHENA trial had to be prioritised over the SWAT, with requirements placed on the practice because of participating in the SWAT minimised. For example, any additional emails about the SWAT were timed to fit in with other communications about the main trial. Qualitative work before intervention development with clinical and non-clinical staff may have resulted in an intervention that was more effective, relevant, and easier to implement.

Finally, despite asking practices to invite receptionists to participate in interviews, there were no expressions of interest from reception staff. However, two interviews were conducted with practice managers, who often have good experience and regular involvement with non-clinical reception staff and therefore their responses are likely to give some insight into experiences of non-clinical staff of the intervention.

### Comparison with existing literature

As far as we are aware, there are no directly comparable studies. Comparing our results to that of other SWATs intended to improve participant recruitment into host trials, a 2023 study examined recruitment into surgical trials following additional staff training. There was no improvement in trial recruitment rates; the authors attribute this lack of effect to poor staff engagement with the intervention and low prevalence of conditions being treated in the host trials, therefore no effect could be demonstrated.^18^

More generally, the age and sex characteristics of patients in our study are comparable with previous studies, based on large national datasets.^19^^,^^20^ A 2014 New Zealand study using a small primary care dataset found that 33% of patients were seen within 3 days of rash onset, and 26% between 3 and 7 days.^21^ Furthermore, in a 2012 UK study, 58.1% of incident cases of shingles received an antiviral prescription, and 97.7% of these antivirals were issued on the day of diagnosis.^22^ Assuming that NICE guidance was followed for these prescriptions, it can be concluded from their study that >50% of patients were diagnosed with shingles <7 days after rash onset.^22^ Their result is comparable with those of other studies.^23^^,^^24^ However, our results demonstrate a comparably higher proportion of patients were seen within the recommended window; 54.6% (*n* = 200/366) of patients within 3 days and 82.2% (*n* = 301/366) within 6 days, across both intervention and control practices (Table 2).

These discrepancies may be the result of our SWAT being carried out after the COVID-19 pandemic and the required transition to remote consulting. The continuing opportunity for consultations to be conducted with accompanying photographs of any rash may have improved access and reduced time to diagnosis for patients with shingles. Furthermore, previous studies found the level of deprivation to affect time to seeking assessment; Forbes *et al* report 58.4% of patients with shingles received antiviral medications in the least deprived areas versus 54.6% in the most deprived (*P*<0.01).^22^ Our study was carried out in relatively affluent practices where the baseline proportion of patients seen within 144 h of rash onset may have therefore been higher. The impact of our intervention may, as a result, have been minimised by this.

### Implications for research and practice

Our study demonstrates that practice-wide educational interventions can be undertaken successfully with remote monitoring. It also provides insight into how behaviour of the non-clinical gatekeeper role affects patient outcomes and if this is modifiable by educational interventions. Our findings also suggest that the most successful forms of education delivery are educational posters or videos. However, our qualitative results indicate that attempting to improve receptionist knowledge to result in improved clinical care for patients, and improved clinical trial recruitment, is unlikely to be successful in a context of under-resourcing and high pressure. Future similar interventions will need to take this into account, alongside the advent of remote and online consultations, and a move away from the traditional model of GP receptionists acting as gatekeepers to appointments. Preliminary qualitative work with intended recipients of an intervention could benefit future primary care educational trials to allow modifications to any intervention design to be made early in response to the rapidly changing primary care structure and workforce.

Furthermore, undertaking a SWAT to also improve trial recruitment when the recruitment model is not uniform across all participating centres increases the level of challenge. Future primary care SWATs designed to improve host trial recruitment should ensure varying individual practice structure and organisation regarding patient flow is acknowledged in any intervention design.

Finally, our results suggest the rate limiting steps to a timely diagnosis of shingles is patient delay in presentation and GP capacity, which are future targets for research.

## Data

Anonymous research data will be stored securely on a research data facility. Requests for access to data must be via a written confidentiality and data sharing agreement. The person applying for use of the data will be scrutinised for appropriate eligibility by members of the research team.
